# Psychosis in Epilepsy vs Late-Onset Schizophrenia: A Case Report

**DOI:** 10.7759/cureus.32692

**Published:** 2022-12-19

**Authors:** Jose J Tascon-Cervera, Alba I Crisostomo-Siverio, Cesar Cardenes-Moreno, Juan F Dorta-Gonzalez, Armando L Morera-Fumero

**Affiliations:** 1 Psychiatry, Hospital Universitario De Canarias, San Cristobal De La Laguna, ESP; 2 Internal Medicine/Dermatology/Psychiatry, Hospital Universitario De Canarias, San Cristobal De La Laguna, ESP

**Keywords:** circumstantial speech, hallucination, neuropsychiatry symptoms, late-onset schizophrenia, epilepsy, psychosis, case report

## Abstract

Psychotic disorders can have a primary or secondary origin. Primary psychosis includes pathologies such as paranoid schizophrenia, acute psychotic episodes, schizoaffective disorder, and other chronic psychiatric disorders. However, in secondary psychosis, there is an organic cause that explains the appearance of psychotic symptoms, such as those secondary to the consumption of psychoactive substances or some neurological or systemic diseases. Psychosis in epilepsy falls under secondary psychosis. It may present as hallucinations and delirium reminiscent of some primary psychoses such as schizophrenia. We present the case of a 57-year-old female suffering from temporal lobe epilepsy who developed psychotic symptoms and whose definitive diagnosis was a challenge given the similarities between some alternative diagnoses, mainly between interictal psychosis of epilepsy and late-onset schizophrenia. We also review the relevant literature. We consider that more studies are required to clarify the relationship between epilepsy and psychosis.

## Introduction

Psychotic disorders can be classified in various ways. One of them focuses on its origin, classifying the disorder as primary or secondary. A primary origin means that there is no other pathology or organic cause that explains the episode [1]. However, psychotic symptoms may also appear secondary to other factors such as the consumption of psychoactive substances or some systemic or neurological pathologies like Wilson’s disease, certain encephalitis, certain dementias such as Lewy body dementia, or even epilepsy [1].

Depending on its duration and evolution, international classifications Diagnostic and Statistical Manual of Mental Disorders (DSM)-5 and International Classification of Diseases (ICD)-10 are used, and there are acute psychotic disorders with a marked start and end and recovery, or chronic psychotic disorders. The latter include schizophrenia, delusional disorder, or schizoaffective disorder. Psychotic disorders usually appear in the third decade of life [2]. Similarly, it is possible for psychotic issues to appear spontaneously, i.e., with a primary origin, with symptoms resembling those observed in schizophrenia in adults aged 40-60 years (late-onset psychosis) or even in those over 60 years (very late-onset psychosis) [3]. 

Psychosis of epilepsy refers to forms of psychosis secondary to epilepsy and are classified according to their temporality in relation to epileptic seizures [4]. According to the literature, there is a predisposition to presenting psychotic symptoms that varies between 19% and 80% over life in epileptic patients [5], with an incidence of 6-12 times greater than in the general population [6].

Some forms of epileptic psychosis may be reminiscent of chronic psychosis such as schizophrenia. This can result in diagnostic mistakes. In this article, we present the case of a 57-year-old female suffering from focal epilepsy of the temporal lobe and admitted to a medium-long stay psychiatric unit, with possible diagnoses being epileptic psychosis and late-onset psychosis. 

## Case presentation

A 57-year-old female, divorced with two children, was brought to the emergency department by the emergency services. She presented behavioral changes at home, causing a fire and harboring delusions of harm by neighbors. Her medical history included epilepsy diagnosed at 27 years of age, which was treated with carbamazepine and phenobarbital. She had no relevant psychiatric history. Four years ago, she left her job and her children started living with their other parent.

In the emergency department, a general analysis was performed, with no significant alterations. Levels of carbamazepine and phenobarbital were measured within the therapeutic range. A head CT was performed, which showed no significant alterations. Once the acute organicity of the condition was ruled out, the patient was admitted to the psychiatric ward.

Four years ago, the patient began to develop ideas of harm focused on the neighborhood, as well as olfactory, tactile, and auditory hallucinations that she experienced with significant discomfort. She said that neighbors had tried to harm her by transmitting odors and vibrations that penetrate the walls and windows. She presented with circumstantial speech and showed a preserved personality, adequate affective resonance, and no negative symptoms. She was started on antipsychotic treatment with paliperidone (9 mg/day) and olanzapine (10 mg/day) and it was decided to consult neurology. An EEG was performed. It showed voltage spikes mostly at the lateral temporal level. These findings were compatible with focal epilepsy with known temporal lobe semiology.

A month after admission, during which time there were no signs of psychopathology improvement, with the tactile hallucinations and delusional ideas of harm and damage persisting with full conviction and increased reactive anxiety, it was decided to transfer her to the medium-long stay ward.

In this ward, it was decided to modify the pharmacological treatment, changing from oral paliperidone to intramuscular at a monthly dose of 150 mg to ensure therapeutic adherence. Olanzapine treatment was initially maintained. There were no side effects. The evolution of the disorder was adequate since the patient gradually distanced herself from the delusional ideas mentioned, with the hallucinations disappearing completely. In addition, she recovered her usual level of appetite and actively participated in the ward’s activities (occupational therapy, psychoeducation, therapeutic outings), showing herself to be effectively euthymic and correctly adapted. Given the good response, the olanzapine was gradually reduced, and then fully suspended a month and a half after admission with no relapses or appearance of new psychopathologies. She agreed to follow up after discharge from ambulatory mental health support.

With family support from her children and ambulatory mental health support, her discharge from the unit was decided with adequate plans for the future and appropriate adherence and tolerance to the treatment scheduled. At the time of discharge, she was conscious, oriented, and collaborative. The delusions that resulted in her admission were distanced. There were no hallucinations, no insomnia, no affective symptoms, or other relevant psychopathology.

She was discharged with the diagnoses of paranoid schizophrenia (ICD-10 F20.0) and temporal focal epilepsy (ICD-10 G40.2).

## Discussion

We discussed a psychotic episode in a female in her sixth decade of life, with no relevant psychiatric history. Given the patient’s characteristics and personal history, we considered the main alternative diagnoses of: (i) a psychotic state secondary to her underlying epilepsy, or (ii) a late-onset psychotic disorder of primary origin.

Various epidemiological studies demonstrate a high predisposition to the development of psychosis in patients diagnosed with epilepsy [5,7]. The risk of developing psychosis would be mainly related to the type of epileptic syndrome, the response to treatment, and the psychosocial conditions [5]. A prospective Finnish trial studied the comorbidity of psychiatric and organic disorders based on the monitoring of a sample of 11017 patients over 28 years, concluding, among other results, a strong link between epilepsy and schizophrenia [8]. Psychosis in epilepsy is usually classified according to its relationship with the time of seizures. Therefore, a study differentiates between two types of psychosis, peri-ictal and interictal [6]. Peri-ictal psychoses are closely linked to epileptic seizures; they are pre-ictal if they occur before the seizure, ictal if they occur during the seizure, and postictal if they occur after the seizure. Interictal psychoses do not have a temporal link with seizures [9]. Both postictal psychosis and interictal psychosis can appear independently in the same type of patient, known as bimodal psychosis. It has been discussed if these types of psychosis are the same entity with different clinical forms [5].

Interictal psychosis is also called psychosis of epilepsy similar to schizophrenia or schizophrenia-like, since from a clinical point of view it usually presents in a similar way to schizophrenia, with this sometimes being the differential diagnosis [5,7]. Therefore, in terms of psychotic semiology, paranoid delusions and auditory hallucinations in the form of voices prevail [7]. In our case, the patient presented auditory and other types of hallucinations as well as delusional ideas of threat from her neighbors.

Various studies have shown clinical differences between interictal psychosis and schizophrenia, which help with the differential diagnosis. Therefore, while it is common in schizophrenia to differentiate between positive (delusions and hallucinations) and negative (affective flatness and anhedonia) semiology, in interictal psychosis, positive symptoms prevail, better preserving personality traits [10]. In our clinical case, negative symptoms were not assessed and the patient’s personality was preserved. In addition, the prognosis of interictal psychosis appears to be better than schizophrenia. This could be due to the tendency of the psychotic symptoms experienced to subside over time [11]. Along with this, good premorbid functioning and lower cognitive deterioration (especially in executive functions and verbal memory) are observed in comparison with schizophrenia [7]. It is known that there is a greater incidence of psychosis similar to schizophrenia in patients with temporal lobe epilepsy [9,12]. In the case presented, the EEG performed on the patient showed findings compatible with focal epilepsy in the temporal lobe. The psychotic semiology characteristic of temporal lobe epilepsy is olfactory hallucinations. The smell most commonly associated with temporal lobe epilepsy is that of burnt leather [12]. In our case, the patient reported interictal olfactory and tactile hallucinations. It is known that somatic delusions can be secondary to olfactory hallucinations in patients with no other history of psychosis [12]. The combination of antiepileptics and antipsychotics for the treatment of patients with psychosis in epilepsy has become widespread. However, it should be noted that there are antiepileptics that increase hepatic metabolism, which reduces the effective levels of antipsychotics [6]. Some authors suggest the use of anti-epileptics with renal metabolism, such as levetiracetam [6].

The other possible diagnosis is a primary psychotic disorder not related to her epilepsy. In this case, this was a late-onset chronic psychotic disorder. Therefore, we also considered a possible diagnosis of late-onset schizophrenia.

Firstly, analyzing the clinical presentation presented, with a clear auditory, tactile, and olfactory hallucinatory component, as well as delusions of damage and harm that generated significant emotional and behavioral repercussions over time, we consider adult-onset paranoid schizophrenia as a diagnosis [3]. Schizophrenia usually has its onset in adolescence or at around 20 years of age, with its presentation being slightly later in women [2]. For this reason, it is also known as early-onset schizophrenia (EOS). However, in approximately 0.1-0.5% of cases, it can appear in older adults [13]. If it appears after the age of 40, it is called late-onset schizophrenia; while if it occurs in patients over 60 years of age it is called very late-onset schizophrenia [13].

Late-onset schizophrenia occurs more frequently in females [3]. In addition, risk factors include being single, social isolation, schizoid or paranoid premorbid personality, or neuropsychological disorders related to the frontal and temporal lobes among others [9]. Our case includes some of these factors; the patient is a female, divorced, in a situation of social isolation, with focal epilepsy of the left temporal lobe, and we cannot rule out the presence or absence of a schizoid or paranoid premorbid personality, although her family described her as a “reserved, secretive, and sometimes distrustful person”. Regarding the symptoms presented, many symptoms are reminiscent of schizophrenia that starts in the first decades of life. In fact, some studies have concluded that there are no clear differences between both pathologies [14]. However, other research has suggested some small differences. As an example, for outpatients, there has been talk of less serious psychotic symptoms in late-onset schizophrenia than in early-onset schizophrenia [15]. However, in hospitalized patients, more serious thought disorders have been observed in late-onset schizophrenia [16]. In addition, it has been suggested that patients with late-onset schizophrenia have presented highly structured persecutory delusions, although with less affective flatness and fewer negative symptoms [17]. This data concurs with our clinical experience in this case, in which very structured delusion with full conviction can be assessed.

Finally, considering the clinical picture presented, with alterations in sensoperception and delirium of systematized harm evolving over several years in a female in her sixth decade of life, she was discharged with the diagnosis of a chronic psychotic disorder. However, there are doubts as to whether the patient actually presented interictal psychosis secondary to her underlying epilepsy. We have the epileptical focus in the temporal lobe [18], the absence of negative symptoms [10], olfactory hallucinations [12], and the duration of the psychotic symptoms [19]. Also, a higher prevalence of psychosis has been reported in epilepsy [6,7] and epilepsy in psychosis [20]. This means that only her evolution on an outpatient basis will allow us to determine the definitive diagnosis.

## Conclusions

This article highlights the difficulty of diagnosing some types of psychotic disorders, given that some aspects such as symptoms or chronicity can overlap, and the prevalence of psychosis in epilepsy and epilepsy in psychosis is increased. In our specific case, we doubt whether this is a primary chronic psychotic disorder like schizophrenia or another type of secondary psychosis such as interictal psychosis in epilepsy. The use of ambulatory EEG could help to determine a long-term correlation between epilepsy and schizophrenia. We think it would be interesting to have more studies that could help clarify the link between epilepsy and psychosis.

## References

[1] Keshavan MS, Kaneko Y (2013). Secondary psychoses: an update. World Psychiatry.

[2] Kessler RC, Amminger GP, Aguilar-Gaxiola S, Alonso J, Lee S, Ustün TB (2007). Age of onset of mental disorders: a review of recent literature. Curr Opin Psychiatry.

[3] Suen YN, Wong SM, Hui CL, Chan SK, Lee EH, Chang WC, Chen EY (2019). Late-onset psychosis and very-late-onset-schizophrenia-like-psychosis: an updated systematic review. Int Rev Psychiatry.

[4] Kanner AM, Rivas-Grajales AM (2016). Psychosis of epilepsy: a multifaceted neuropsychiatric disorder. CNS Spectr.

[5] González Mingot C, Gil Villar MP, Calvo Medel D (2013). Epileptic peri-ictal psychosis, a reversible cause of psychosis. Neurologia.

[6] Mendoza-Bermúdez C, Gómez-Arias B (2009). Psychosis in epilepsy (Article in Spanish). Rev Colomb Psiquiatr.

[7] D'Alessio L, Giagante B, Ibarra V (2008). Analysis of psychotic disorders in patients with refractory partial epilepsy, psychiatric diagnoses and clinical aspects. Actas Esp Psiquiatr.

[8] Mäkikyrö T, Karvonen JT, Hakko H (1998). Comorbidity of hospital-treated psychiatric and physical disorders with special reference to schizophrenia: a 28-year follow-up of the 1966 northern Finland general population birth cohort. Public Health.

[9] Pérez-Longás B, Pomares-Martínez T, Pedrós-Roselló A (2017). Late-onset psychosis and Charles Bonnet syndrome: diagnostic difficulties. A case report. Psiquiatr Biológica.

[10] Abreu-de la Torre CL, Pedroso I, García-Navarro ME, Macías-Betancourt R, Cubero-Rego L (2010). Interictal psychosis: presentation of a case (Article in Spanish). Psiquiatria.

[11] Agrawal N, Mula M (2019). Treatment of psychoses in patients with epilepsy: an update. Ther Adv Psychopharmacol.

[12] Gandhi P, Ogunyemi B, MacDonald A, Gadit A (2012). Psychosis in temporal lobe epilepsy: atypical presentation. BMJ Case Rep.

[13] Tampi RR, Young J, Hoq R, Resnick K, Tampi DJ (2019). Psychotic disorders in late life: a narrative review. Ther Adv Psychopharmacol.

[14] Wake R, Miyaoka T, Araki T (2016). Regional cerebral blood flow in late-onset schizophrenia: a SPECT study using 99mTc-ECD. Eur Arch Psychiatry Clin Neurosci.

[15] Brichant-Petitjean C, Legauffre C, Ramoz N, Ades J, Gorwood P, Dubertret C (2013). Memory deficits in late-onset schizophrenia. Schizophr Res.

[16] Huang C, Zhang YL (2009). Clinical differences between late-onset and early-onset chronically hospitalized elderly schizophrenic patients in Taiwan. Int J Geriatr Psychiatry.

[17] Sato T, Bottlender R, Schröter A, Möller HJ (2004). Psychopathology of early-onset versus late-onset schizophrenia revisited: an observation of 473 neuroleptic-naive patients before and after first-admission treatments. Schizophr Res.

[18] Nakahara S, Adachi M, Ito H, Matsumoto M, Tajinda K, van Erp TG (2018). Hippocampal pathophysiology: commonality shared by temporal lobe epilepsy and psychiatric disorders. Neurosci J.

[19] Adachi N, Akanuma N, Ito M, Okazaki M, Kato M, Onuma T (2012). Interictal psychotic episodes in epilepsy: duration and associated clinical factors. Epilepsia.

[20] Chang YT, Chen PC, Tsai IJ (2011). Bidirectional relation between schizophrenia and epilepsy: a population-based retrospective cohort study. Epilepsia.

